# Formulation and Analytical Evaluation of Liquid Cannabidiol Preparations: Comparative Study of Oil-Based Solutions and Emulsions

**DOI:** 10.3390/pharmaceutics17121533

**Published:** 2025-11-28

**Authors:** Robert-Alexandru Vlad, Lénárd Farczádi, Denisa Paliștan, Cezara Pintea, Paula Antonoaea, Emöke-Margit Rédai, Andrada Pintea, Cornelia-Titiana Cotoi, Adriana Ciurba, Magdalena Bîrsan, Ruxandra-Emilia Ștefănescu

**Affiliations:** 1 Pharmaceutical Technology and Cosmetology Department, Faculty of Pharmacy, George Emil Palade University of Medicine, Pharmacy, Science and Technology of Targu Mures, 38th Gheorghe Marinescu Street, 540142 Targu Mures, Romania; 2 Chromatography and Mass Spectrometry Laboratory, Centre for Advanced Medical and Pharmaceutical Research, George Emil Palade University of Medicine, Pharmacy, Science and Technology of Targu Mures, 38th Gheorghe Marinescu Street, 540142 Targu Mures, Romania; 3 Medicine and Pharmacy Doctoral School, George Emil Palade University of Medicine, Pharmacy, Science and Technology of Targu Mures, 38th Gheorghe Marinescu Street, 540142 Targu Mures, Romania; 4 Department of Drug Industry and Pharmaceutical Biotechnology, Faculty of Pharmacy, “Grigore T. Popa” University of Medicine and Pharmacy from Iasi, 16 Universitatii Street, 700115 Iasi, Romania; 5 Department of Pharmacognosy and Phytotherapy, Faculty of Pharmacy, George Emil Palade University of Medicine, Pharmacy, Science and Technology of Targu Mures, 38th Gheorghe Marinescu Street, 540142 Targu Mures, Romania

**Keywords:** cannabidiol, homogenous preparation, emulsions, stability test, analytical assay

## Abstract

**Background/Objectives**: Cannabidiol (CBD) is a non-psychoactive compound found in the *Cannabis sativa* plant. Due to its broad therapeutic potential, CBD is often incorporated into various pharmaceutical formulations. This study aimed to evaluate homogenous (oil-based) and heterogeneous (emulsion-based) liquid preparations of CBD using different fatty oils and provide a comprehensive comparative framework for the development of stable liquid dosage forms of cannabidiol (CBD), with direct applications in pharmaceutical formulations. **Methods**: The oils and emulsions were qualitatively analysed to assess their stability and suitability as CBD carriers. Ultraviolet (UV) spectrophotometry and High-Performance Liquid Chromatography (HPLC) were employed for quantifying CBD in the formulations and also characterising them in terms of product quality. **Results**: The results indicated that sunflower oil is the most stable and analytically compatible matrix, with CBD recovery close to 100% and minimal degradation over time. Conversely, linseed and pumpkin seed oils exhibited significant analytical interference and oxidative instability. Oil-in-water emulsions prepared with a 4% Tween 80/Span 80 mixture demonstrated optimal physical stability and droplet size distribution. **Conclusions**: Overall, both formulations can be regarded as suitable pharmaceutical carriers for CBD delivery.

## 1. Introduction

Cannabidiol (CBD) is a phytocannabinoid extracted from the *Cannabis sativa* plant, characterised by its lack of psychoactive effects and extensively researched for its therapeutic potential. It is included in various pharmaceutical formulations available on the market, such as oily solutions. One example, approved in both the European and United States (US) markets, is Epidiolex/Epidyolex, which contains 10% CBD suspended in an oily solvent and is marketed as a treatment for patients with Lennox-Gastaut and Dravet syndrome who are unresponsive to other active ingredients [1,2,3].

In the European Union (EU), no topical medicinal products containing cannabidiol (CBD) are currently authorised; however, the compound is not listed among the prohibited ingredients in the EU Cosmetic Ingredients Database (CosIng), which has enabled its incorporation into a growing number of cosmetic preparations and specific Class I medical devices. As a result, CBD is a multifaceted active ingredient that can be administered internally and externally. Typically, oily solutions can be administered internally in drops, while emulsions can be used both internally and externally. Furthermore, the emulsions can be incorporated into semi-solid formulations by merging them with a hydrogel, resulting in an emulgel [1,3].

**Figure 1:**
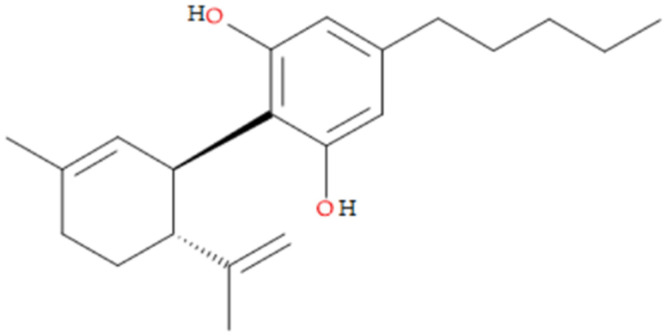
CBD-chemical structure.

Given that this active ingredient falls within the second category of the Biopharmaceutical Classification System (BCS II), its physicochemical properties are critical. CBD can be described as a crystalline, colourless to pale yellow powder with a molecular weight of 314.5 g/mol and the chemical formula C_21_H_30_O_2_. The molecular structure is shown in Figure 1. CBD exhibits high lipophilicity, with a logP value of approximately 6.3, which affects its aqueous solubility (estimated to be less than 1 µg/mL) and oral bioavailability. Consequently, due to its low hydrophilicity, the most appropriate pharmaceutical formulation for CBD is an oily solution, which has already been trademarked and is commercially available. CBD contains two phenolic hydroxyl groups, indicating weak acidic properties (pKa = 9.13). It is practically insoluble in water but dissolves in organic solvents such as ethanol and specific liquid lipids. Its chemical stability depends on the formulation type, storage conditions—including light exposure, temperature, and oxygen exposure—and pH levels; an alkaline pH increases the risk of degradation [3].

Due to its increased lipophilicity, two of the most common pharmaceutical formulations in which the proposed active ingredient will be incorporated are oily solutions and emulsions, both of which are discussed below.

CBD oils are influenced by environmental factors such as light, oxygen, and temperature, with elevated temperatures accelerating the degradation of the active compound and consequently reducing shelf life. Numerous studies have been conducted to evaluate the stability of CBD oils at different concentrations (5% and 10%) across various types of oils, with cannabis oil among the most frequently used [4]. Prior research has indicated that despite labels asserting the absence of cannabinoids in cannabis oils, certain instances have detected a cumulative concentration exceeding the labelled amount. Consequently, an alternative oil that does not contain or derive from the cannabis plant is required to mitigate the risk of cannabinoid accumulation and the associated potential for adverse effects. Given that the active ingredient possesses a low melting point, it is presumed to be less stable than compounds with higher melting points. Maintaining a lower temperature and storing the oil under controlled conditions may enhance the stability of these lipid-based solutions [5,6,7,8,9]. Furthermore, oil-based systems enhance dermal and mucosal permeation of this highly lipophilic molecule, thereby improving therapeutic efficacy. Although advanced technologies such as nanoemulsions or micellar systems can, in principle, enable aqueous-based CBD gels, these require complex surfactant mixtures and processing steps, making simple oil-based solutions the most robust and pharmaceutically feasible approach [10,11].

CBD emulsions serve as delivery systems designed to enhance the low hydrophilicity and poor oral bioavailability of CBD. These pharmaceutical formulations are produced by dissolving active ingredients in a lipid phase and then dispersing them as fine droplets within an aqueous phase. The addition of lipid components into the hydrophilic phase is facilitated by emulsifiers with different hydrophilic–lipophilic balance (HLB) values. Oil-in-water (O/W) emulsions are generally preferred for oral use because they improve patient palatability (i.e., higher patient acceptability); additionally, droplet size can enhance biopharmaceutical and pharmacokinetic properties. Incorporating CBD into this heterogeneous mix provides greater stability during storage under atmospheric conditions; furthermore, exposure to light and oxygen has a less significant impact on the active ingredient compared to an oily solution [10,11,12]. Emulsions provide several advantages, making them suitable for CBD delivery, such as:

- thermodynamic stability,
- small particle size that increases the surface area for absorption, enhancing the dissolution rate and bioavailability of CBD,
- allows the simultaneous incorporation of lipophilic ingredients (such as CBD) and hydrophilic excipients (or other active ingredients that outline an increased hydrophilicity),
- improve formulation flexibility,
- increase the therapeutic efficacy,
- can be tailored for various administration routes—oral or topical—offering versatility for pharmaceutical and cosmetic applications [13,14,15,16].

Bonn-Miller and his collaborators outlined that oily formulations are generally less likely to be mislabelled compared to vaporisation liquids, which are the most frequently mislabelled. The same study showed that more than 40% of the products tended to be undervalued, and more than 30% were overlayered [8].

In a previously published study, it was observed that the cannabis oil contained a small amount of CBD (<0.5%), even though the label stated that the CBD oil contained no CBD or tetrahydrocannabinol (THC) [4]. As a result, another oil should be used to develop CBD oil that will not lead to an over-labelled product. Since CBD is a lipophilic ingredient, the initial formulation chosen is an oily solution. Practically, the previously obtained results served as the basis for this study.

de Aquino et al. developed two distinct nanoemulsions, one containing CBD and the other containing tetrahydrocannabinol (THC), to reduce brain damage and treat convulsions in mice treated with pentylenetetrazole. By using a small amount of oily phase (about 10%), they obtained very stable emulsions, characterised by low particle size (<250 nm) and a low Polydispersity Index (<0.1), which highlights the increased homogeneity of the pharmaceutical formulation [10].

Demisli et al. developed nanoemulsions with CBD at concentrations ranging from 0.1% to 0.5%. Since the concentration range was narrow, the amount of active ingredient did not significantly affect the particle size (in both cases, particles smaller than 130 nm were observed on the day of preparation). During the stability study, it was observed that over time, the particle size tended to increase while the system remained stable with a PdI < 0.25 for all determinations conducted [12].

In both cases, the CBD concentration was below 1%, depending on the administration route and the target population (mice). In contrast, this study employed a higher CBD concentration, exceeding 4%, which has a therapeutic effect in humans, in the liquid formulations. Additionally, it is well established that using a larger amount of active ingredient generally increases the droplet size. For oils, a concentration of 10% (*w*/*w*) was considered, which is equivalent to the product Epidiolex, marketed across the United States and in some European countries. Practically, based on previous experience with CBD oils, new vehicles were evaluated to select the one that is most stable from an analytical and technological perspective. The most stable oil will be identified using a mathematical scoring system: control oils and CBD oils will receive scores, which are summed to determine the oil with the best properties based on the parameters evaluated. Furthermore, the oil with the highest score will be incorporated in a emulsion. For this heterogenous formulation, the preparation methods (conventional or automated) and the amount of emulsifier were varied to evaluate their effects on the emulsion’s properties and quality. The oils can be administered in drops, since the patient cannot use them in high amounts due to the acceptability issues, while the emulsion outlines a better palatability and can be used internally and administered by using other measurement methods (graduated spoon, syringes).

## 2. Materials and Methods

### 2.1. Chemicals and Reagents

The following chemicals and reagents were used throughout this investigation: cannabidiol (CBD) with a purity of 99.5%, supplied by Trigal Pharma (Vienna, Austria); refined sunflower oil from Argus S.A. (Constanța, Romania); cold-pressed pumpkin seed oil and linseed oil obtained from S.C. Dachim S.R.L. (Turda, Romania); and sesame oil provided by Auchan (Bucharest, Romania). The emulsifiers included Tween 80 (polyoxyethylene sorbitan monooleate), supplied by Sigma-Aldrich (Milano, Italy), and Span 80 (sorbitan monooleate) from Carl Roth GmbH (Karlsruhe, Germany). Vitamin E (α-tocopherol) was sourced from Fagron (Trikala, Greece). Simultaneously, Cosgard (a mixture of benzyl alcohol and dehydroacetic acid) was acquired from MAYAM (Elemental, Oradea, Romania, originating from the USA). HPLC-grade acetonitrile and methanol were obtained from Honeywell (Raunheim, Germany). Ethanol, ethyl ether, chloroform, glacial acetic acid, and potassium iodide were procured from Merck KGaA (Darmstadt, Germany). All aqueous solutions were prepared using ultrapure water supplied by Q Test S.R.L. (Iași, Romania). All chemicals and solvents used in this study were of an analytical grade.

### 2.2. Physicochemical Evaluation of Fatty Oil Vehicles

Density, acid value, saponification value, peroxide value, and ester value were determined according to the methods described in the European Pharmacopoeia 11th Edition (Ph. Eur. 11) [17].

#### Organoleptic Properties

The oils were evaluated for colour, odour, and appearance.

### 2.3. Preparation of Homogenous Oil-Based CBD Formulations

Four oil-based formulations were prepared by dispersing CBD into different fatty oils at a final concentration of 10% (*w*/*w*). The oils used included refined sunflower oil, cold-pressed pumpkin seed oil, linseed oil, and sesame oil. CBD was added to these oils by manual stirring, then homogenised for 30 min with a magnetic stirrer MS-H280-Pro DLAB agitator (DLAB, La Mirada, CA, USA). The resulting oils were labelled U1–U4, corresponding to: U1—sunflower oil, U2—pumpkin seed oil, U3—linseed oil, and U4—sesame oil.

#### 2.3.1. Short-Term Stability Study

The amount of CBD was determined using a previously validated chromatographic method (HPLC), with the analytical performance assessment detailed in Section 2.5.2 [4,5]. The active pharmaceutical ingredient (API) was analysed at 1 month (T1), 2 months (T2), and 3 months (T3). Additionally, the presence or absence of fungal filaments was assessed during the organoleptic study at the specified time points.

#### 2.3.2. Oil Selection—Mathematical Scoring

To select the best oil, the properties of various oils, including CBD oils, were evaluated, resulting in a maximum score of 10 for each oil. The parameters considered are listed below, with their mathematical scoring detailed in Table 1. For each parameter, if the results are close to the expected value, the parameter receives the highest score (2). To differentiate the results, different ranges were considered, with each range assigned a different score. For blank oils, the parameters assessed included density, acid index, saponification index, peroxide index, and ester index. For CBD oils, the evaluation focused on the assay method, organoleptic properties (colour, smell, appearance), and stability study. The maximum score per category of oils was 10, obtained by summing the scores for each parameter. To select the most suitable oil for the development of the emulsion, the scores for both the blank oil and CBD oil were combined (with a maximum total of 20). Ultimately, the oil with the highest overall score was chosen for incorporation into the emulsion.

### 2.4. Emulsions with or Without an Active Pharmaceutical Ingredient Loaded

Eight emulsions without CBD were prepared using sunflower oil with varying amounts of surfactants (Tween 80 and Span 80) at different concentrations: 3%, 4%, 5%, and 10% (*w*/*w*). Two preparation methods were employed: a conventional method using a mortar (E_m_) and an automatic method utilising a magnetic stirrer operated at 500 rpm for 30 min (E_a_). For both, the following parameters were considered: emulsification temperature (room temperature), mixing time (10 min), and stirring rate (500 rpm only for the automatic method). The emulsions were designated as follows: E_3%m_–E_10%m_ for the conventional method and E_3%a_–E_10%a_ for the automatic mixing method (see Table 2). Considering the specific Hydrophilic–Lipophilic Balance (HLB) values of the selected surfactants and the optimal HLB value (10), the percentages of each emulsifier were determined and tested. E_CBD_ and E_blank_ were prepared after the stability test was conducted, based on the best results for the amount of emulsifiers used. To prevent oxidation of the active ingredient, vitamin E was added to the aforementioned formulation, while Cosgard was used to preserve the emulsion. The latter two ingredients were only included in the final formulations prepared after the stability test.

#### 2.4.1. Preparation Steps

For the emulsions without API, the lipophilic emulsifier (Span 80) was dispersed in sunflower oil, while the hydrophilic one (Tween 80) was dispersed in ultrapure water. Equal amounts of oily and hydrophilic phases were mixed in a mortar to produce the emulsions labelled E_3%m_–E_10%m_, prepared using a conventional method. In the second method, the hydrophilic and lipophilic components were added to a Berzelius glass and mixed for 30 min at 500 rpm (Table 3). For the latter two formulations mentioned in Table 2, vitamin E and Cosgard were dispersed in the lipophilic mixture.

The hydrophilic–lipophilic balance (HLB) of Tween 80 and Span 80 is 15 and 4.2, respectively. The optimal HLB for sunflower oil is 10, and the required emulsifier ratio was calculated using the Pearson square method.

#### 2.4.2. Establishing the Emulsion Type

Method 1: Dilution method. Two samples of 1 mL each were diluted with water (the first) and oil (the second), and the behaviour of the emulsion was observed. If the emulsion can be diluted with water without showing any phase discontinuity, it can be considered O/W. Conversely, if the emulsion can be diluted with oil, a W/O emulsion is obtained, in accordance with Bancroft’s stipulations [18].

Method 2: Colouring Technique. In two separate test tubes, 1 mL of emulsion was combined with 1–2 drops of a hydrophilic dye (1% *w*/*w* Methylene Blue) in the first tube and with a lipophilic dye (Sudan III) in the second. If a uniform colouration appears upon the addition of Methylene Blue, the emulsion is identified as oil-in-water (O/W). Conversely, if a homogeneous mixture is observed with Sudan III, the emulsion is classified as water-in-oil (W/O).

The two methods can be used simultaneously to compare and validate the results.

#### 2.4.3. Stability Index Evaluation

To determine the influence of the preparation method and the amount of emulsifiers used, 5 mL of each emulsion was poured into a 5 mL glass cylinder, and the water volume was measured after 1-, 2-, and 24 h following preparation. Using Equation (1), the stability index was calculated.

(1) $$S\%=\frac{V1-V2}{V1}\times 100$$

where:

S%—stability index;
V1—theoretical water volume;
V2—practical water volume.

#### 2.4.4. Particle Size Evaluation

To evaluate this parameter, 30 particles were examined for size using an electron microscope (Optika Microscopes, Ponteranica, Italy). The emulsion was agitated, and a single drop was analysed for size; 30 oil drops were evaluated. The mean size was calculated and expressed as the average ± standard deviation (SD). Additionally, the following parameters were considered during the assessment: the minimum particle diameter (dmin), the largest particle diameter (dmax), the frequency of drops within an interval (f), the mean diameter of the interval (dm), and the percentage of drops within the interval (n%). Ranges for ten particles were considered for both the CBD emulsion and the emulsion without active ingredients. A frequency curve was plotted.

### 2.5. Analytical Determinations of CBD Preparations

Two analytical techniques were employed to determine the CBD content in the proposed liquid formulations: a UV-spectrophotometric method and an HPLC method. For each technique, standard performance was assessed, and both were utilised after achieving satisfactory linearity and selectivity parameters.

#### 2.5.1. Spectrophotometric Quantification of CBD in Oily Solutions

A stock solution of 1 mg/mL CBD in acetonitrile was prepared. From the stock solution, a calibration curve was prepared in the range of 0.5–25 µg/mL. CBD oils were diluted 1:100 (*v*/*v*) with acetonitrile. The active ingredient was assessed at 208 nm in a 1 cm-diameter glass cuvette.

A calibration curve (R^2^ > 0.99) was generated at 208 nm. The CBD emulsions were diluted to achieve a concentration within the proposed range. Absorbances were measured and compared to the standard curve.

#### 2.5.2. UHPLC Analysis of CBD Oils and Emulsions

High-performance liquid chromatography (HPLC) was used to assess the uniformity and stability of CBD levels in various vegetable oils. The analyses occurred at the Advanced Medical-Pharmaceutical Research Centre, within the Chromatography and Mass Spectrometry Laboratory (CROMS) of George Emil Palade University of Medicine, Pharmacy, Science and Technology of Targu Mures. A UHPLC Flexar (PerkinElmer, Shelton, CT, USA) system and a Gemini NX C18 column (3 µm, 100 × 3 mm) were used to quantify CBD in the prepared oils, following a method previously developed and validated by the research group [16].

A 1 mg/mL stock solution of CBD was prepared, and seven standard dilutions were then ready to assess the method’s linearity. Linearity was evaluated at three time points after preparation: 1 month (T1), 2 months (T2), and 3 months (T3). Other specific parameters are included in the Supplementary Materials.

Each oil sample was diluted twice: 1:100 and 1:20. For the 1:100 dilution, 100 µL of the sample was mixed with acetonitrile in a 10 mL volumetric flask. From this, 500 µL was further diluted to 10 mL (1:20). Blanks (non-CBD oils) were prepared in the same manner.

Samples and standards were filtered and transferred into HPLC vials. The mobile phase consisted of a 30:70 (*v*/*v*) mixture of water and acetonitrile, and the detection wavelength was set to 208 nm. The injection period lasted 3.5 min under a pressure exceeding 200 bars.

### 2.6. Statistical Evaluation

Statistical analysis of the CBD assay in the emulsion was performed using the HPLC method in GraphPad Prism 10 (GraphPad Software, Boston, CA, USA). The ROUT test was used to identify and exclude outliers at the Q = 1% level, followed by the Shapiro–Wilk normality test. Since only two datasets were compared, the Student’s *t*-test was applied. Results are presented as mean ± SD. To assess the quality index, a one-way analysis of variance (ANOVA) was conducted, followed by the post hoc Tukey test. Statistically significant differences between samples are indicated by different letters. The significance level was set at 0.05 (*p*).

## 3. Results and Discussion

This section presents the results and discussion for both CBD oils, as well as the procedures followed to select the most stable CBD oil for incorporation into a CBD emulsion, along with its pharmaceutical and technological characterisation.

The choice of refined sunflower oil, cold-pressed pumpkin seed oil, linseed oil, and sesame oil as lipid carriers was based on their wide availability, proven safety profile, and everyday use in pharmaceutical and nutraceutical products. These edible vegetable oils offer suitable lipophilic environments for dissolving cannabidiol (CBD) and vary in their fatty acid profiles and natural antioxidant contents (tocopherols in sunflower oil, sesamol in sesame oil), factors known to affect oxidative stability and compatibility with analytical methods. Additionally, using non-cannabis-derived oils reduces the risk of uncontrolled cannabinoid levels and regulatory issues, while allowing for a systematic comparison of physicochemical performance for developing stable CBD liquid dosage forms.

### 3.1. Physicochemical Evaluation of Fatty Oil Vehicles

In addition to density, the quality indices (acid value, saponification value, peroxide value, and ester value) were evaluated to determine which oil would be used to develop the CBD oils and emulsions.

#### 3.1.1. Density Evaluation

For each fatty oil, the density was measured and is shown in Table 4. Minor differences were observed between the oils, all of which were very close to the Ph. Eur. 11 requirements, as shown in Table 4 [18]. All oils recorded density values lower than the Ph. Eur. 11 standards, possibly due to the method used (the picnometric one), since digital densitometers are considered to have a higher accuracy [17]. Additionally, the deviation from the standards was less than 5%; therefore, all oils received a perfect score of 2 for this parameter.

#### 3.1.2. The Quality Index Assessment

**Figure 2:**
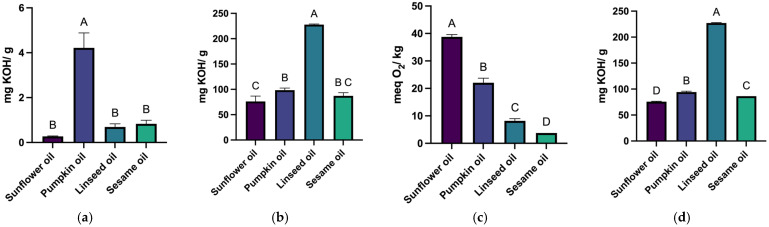
Acid value (**a**), saponification value (**b**), peroxide value (**c**), and ester value (**d**) were determined for the four fatty oils. Different letters above columns indicate a statistically significant difference at *p* < 0.05.

The results obtained in the physicochemical evaluations of the fatty oils are underscored in Figure 2.

Table 5 highlights the results, considering the evaluated indices and the Ph. Eur. 11 stipulations [17].

Sunflower oil had the lowest acid value (0.27 mg KOH/g) and the highest peroxide value (38.78 meq O_2_/kg). Linseed oil showed the highest saponification value (227.82 mg KOH/g). Ester values were highest in linseed oil (227.13 mg KOH/g) and lowest in sunflower oil (75.78 mg KOH/g).

Close attention should be paid to sunflower oil, as this solvent exhibited the highest peroxide value and the lowest acid value. Since the peroxide value is high at the start of the evaluation, the active ingredient might undergo accelerated oxidation. To test this possibility, a three-month stability study will be conducted to assess the risk of CBD decomposition.

For future studies, the oil source and freshness should be considered, as they might imply a low peroxide level. Another option to consider is using neutralised sunflower oil, which has a very low acid index (<0.2). For this excipient, which is usually used for parenteral administration, the peroxide value should be assayed to determine the oil’s initial stability.

Four different fatty oils were used to prepare oil-based solutions containing 10% cannabidiol (CBD). These formulations underwent physicochemical quality assessments through a series of standard analyses. The acid value indicates the concentration of free fatty acids and serves as an indicator of the oil’s quality. For sunflower oil, the results show that the acid value complies with the Ph. Eur. 11 standards (0.5 mg KOH/g) [17]. For pumpkin seed oil, the literature reports a maximum acceptable acid value of 1.32 mg KOH/g. The experimental value obtained in this study exceeded this limit, suggesting that the oil may have undergone degradation or was produced by heat extraction followed by refining, despite product labelling claiming cold-press extraction. For linseed oil, the acid value determined was 0.69 mg KOH/g. The Ph. Eur. 11 specifies a maximum acid value of 4.5 mg KOH/g for *Lini oleum virginale* [17]. The results obtained in the present study are within the acceptable range, indicating a favourable quality profile and an optimal level of free fatty acids. Regarding sesame oil, the Ph. Eur. 11 limits the maximum acid value at 0.5 mg KOH/g [17]. The value obtained was slightly above this limit, suggesting a mildly elevated free fatty acid concentration, possibly due to minor oxidative degradation.

The saponification value functions as an indicator of the average molecular weight of fatty acids and bears an indirect correlation with both foaming properties and emulsion behaviour. The sunflower oil sample exhibited a saponification value of 76.05 mg KOH/g, markedly below the expected range of 186–194 mg KOH/g. This observation suggests a diminished foaming capacity and may imply the presence of longer-chain fatty acids or a reduced ester content. Pumpkin seed oil demonstrated an experimental value of 98.39 mg KOH/g compared to a theoretical value of 181.76 mg KOH/g. This discrepancy indicates incomplete saponification or insufficient extraction duration. Linseed oil exhibited a saponification value marginally above the upper limit of the theoretical range (188–195 mg KOH/g), potentially indicating a higher concentration of short-chain fatty acids and an enhanced foaming potential. Furthermore, sesame oil showed a value lower than anticipated, relative to the literature range of 188–193 mg KOH/g, suggesting either limited saponification capacity or the need for an extended reaction period.

Peroxide value is an essential indicator of lipid oxidation and the presence of peroxidised lipids. According to Ph. Eur. 11, the maximum allowed peroxide value for sunflower oil is 10 meq O_2_/kg [17]. The experimental result exceeded this limit, indicating non-compliance and possible oxidative degradation. For pumpkin seed oil, the literature reports a maximum theoretical value of 2.02 meq O_2_/kg. The high peroxide value obtained suggests poor oxidative stability. In contrast, linseed oil showed a peroxide value within the acceptable range (≤15 meq O_2_/kg) according to Ph. Eur. 11, confirming its adequate oxidative stability under the tested conditions [17]. Sesame oil exhibited a slightly higher peroxide value compared to the theoretical maximum of 2.34 meq O_2_/kg. However, the increase was not significant, indicating acceptable oxidative stability and a suitable fatty acid composition.

These parameters are essential not only for establishing the pharmaceutical quality of the lipid vehicles but also for predicting their behaviour in terms of stability and interaction with the active compound (CBD), as noted in similar studies on lipid excipients in drug delivery systems [17,19,20,21].

Based on the overall physicochemical assessment, refined sunflower oil was identified as the most suitable lipid vehicle for CBD due to its low acid value within pharmacopoeial limits, an acceptable ester and saponification profile, and superior analytical compatibility, despite presenting a higher peroxide value that can be mitigated by controlled storage conditions.

The deviations observed in this article may indicate potential quality issues, such as oxidative degradation or inconsistencies in sourcing and handling. The selected oils were food-grade quality (not pharmaceutical-grade, which might explain the saponification and ester values that were out of range). To investigate and explain the out-of-range results, future work should include evaluating storage conditions, as factors like light, O_2_, and temperature can cause alterations in the oils and affect GC-MS fatty acid profiling, ensuring that the oils meet their standard compositional benchmarks.

### 3.2. Organoleptic Properties of the Unloaded Oils and CBD Oils

The sunflower oil displayed the following characteristics: clear yellow colour, transparent liquid (fluid at room temperature), with an almost imperceptible (neutral) odour. The addition of CBD did not change the properties mentioned earlier [22].

The pumpkin oil was a dark green colour, a clear liquid with a pleasant nutty odour, and was usually liquid at room temperature. By mixing the CBD with the pumpkin oil, the organoleptic properties remained unchanged [23,24].

Linseed oil is a golden-yellow oil with a mild nutty aroma when fresh. It is smooth, light, and fluid, but can become bitter and develop a strong odour if it oxidises [25]. The dissolution of CBD in linseed oil did not affect the organoleptic properties evaluated in this study.

Sesame oil is a clear, golden oil with a characteristic nutty aroma, more intense when roasted. It is smooth and fluid, adding a distinctive flavour to culinary applications [26,27]. By incorporating CBD into this oil, no modifications to the organoleptic properties were observed.

All selected fatty oils met the organoleptic requirements; as a result, they received a perfect score for each parameter evaluated—colour, odour, and aspect—totalling 6.

Based on the results for the unloaded CBD oils, the recommended oils for further use are linseed oil and sesame oil, both of which achieved a cumulative score of 6. In contrast, the other two oils received a score of 4. Given the need for a rapid analytical methodology to evaluate the active ingredient, a cumulative score will be calculated from results for both unloaded oils and CBD oils.

The colour is not a major obstacle for an oral product, but the odour significantly affects the acceptability of an oral solution, especially in paediatric patients. Consequently, some modifications to the formulation, such as adding flavour and sweetener agents, will be necessary. These agents can be incorporated into the lipophilic matrix (for oils: acesulfame potassium, sucralose, erythritol, and xylitol), while in the case of an emulsion, a wide range of agents can be used since all lipophilic and hydrophilic sweeteners are suitable.

### 3.3. Stability Study—CBD Assay

#### 3.3.1. Spectrophotometric Determinations of CBD in Oil Formulations

The theoretical concentration of each oil was calculated and expressed in µg/mL. The percentage deviation between the measured and theoretical concentrations was determined, and a corresponding mathematical score was assigned. In the U2 formulation, pumpkin oil contained an ingredient that interfered with the active compound, leading to higher-than-expected absorbance (peaks overlapped, resulting in a hyperchromic effect). In contrast, for sesame and linseed oils, a hypochromic effect could be observed. Since the oils used in the U2–U4 formulations are chemically complex, comprising triglycerides, pigments (such as chlorophylls and carotenoids), phenolic antioxidants, and oxidation products, there is an increased risk of overlapping absorbance bands in the UV region. Another analytical method is being requested for the evaluation of the active pharmaceutical ingredient content [24,28].

If the spectrophotometric method could be used to assess the proposed active ingredient, an additional point was awarded to that specific oil. From this perspective, only U1 received an extra point, while the other three oils did not earn any points based on the spectrophotometric results; consequently, the active ingredient was further analysed by HPLC.

The results of the spectrophotometric assay for the active ingredient in the proposed CBD oils, along with the percentage deviation from the theoretical concentration, are detailed in Table 6.

#### 3.3.2. HPLC Quantification of CBD in Oil Formulations

Method validation confirmed satisfactory linearity (R^2^ > 0.99), precision, and accuracy (Table S1). Since the spectrophotometric method was not suitable for three out of the four oils, an alternative analytical quantification method was employed, utilising HPLC (a validated method previously used to determine CBD in various matrices, including orodispersible tablets and films, suspensions, hard capsules, oils, and powders). The analytical performance of this method was confirmed beforehand [4,5,29,30].

Calibration curves for different time frames—T1 (after 1 month), T2 (after 2 months), and T3 (after 3 months)—exhibited consistent retention times (~2.45–2.48 min), high peak heights, and areas proportional to concentration. R^2^ values were above 0.99 at all time points (Table S1), confirming method linearity (Figure S1) and selectivity (Figure S2). Other specific parameters obtained during the validation are included in the Supplementary Materials (Table S1).

For a more accurate evaluation, the percentage deviations from the theoretical concentrations were highlighted in green if the results were close to the expected values and in red if they fell outside the acceptable range. As shown in Table 7, a noticeable accumulation also occurred with U2 oil, despite HPLC generally offering better selectivity. However, this was not always the case, as one component at the chosen wavelength (210 nm) interacted with CBD. Although the results for the U3 formulation were closer to the theoretical values, significant deviations remained; additionally, after 3 months, the concentration had nearly halved.

**Figure 3:**
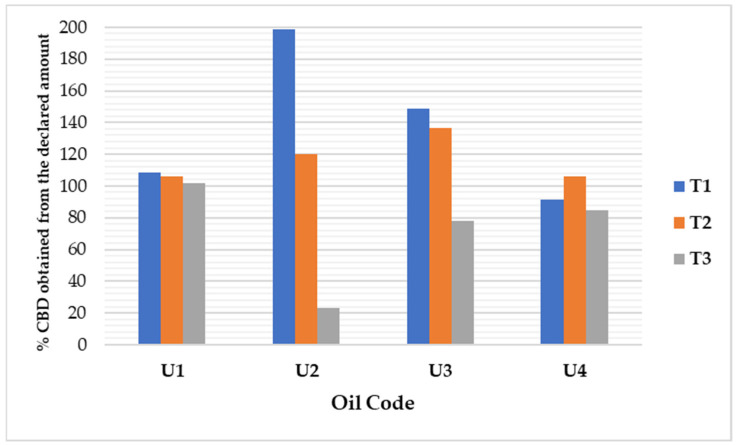
Percentage of CBD assayed at different time-frames (T1—1 month, T2—2 months, T3—3 months).

Sunflower oil (U1) demonstrated the best stability over 3 months at 22 ± 2 °C in the dark (%deviation < 10% for all three timeframes selected). HPLC was confirmed as optimal for CBD quantification in this matrix. Pumpkin oil (U2) experienced significant degradation, with CBD levels decreasing by nearly 90% after three months. Linseed oil (U3) requires post-dilution sonication and centrifugation or replacement with non-polar solvents (e.g., acetone, ethyl acetate) for the accurate quantification of CBD. Sesame oil (U4) remained more stable over time compared to sunflower oil, and HPLC was suitable for its CBD analysis. The results stated in Table 7 are supported by the CBD recovered at different time-frames considered for the stability study outlined in Figure 3.

The stability of the oils was calculated considering the theoretical (expected) result. The results for U4 are good considering the first two determinations (at 1 and 2 months), but at the last evaluation, the difference from the expected result was higher −15% and automatically received a lower score. If the results obtained after 1 month of storage had been compared with the ones obtained after 1 month, the final score would have been different.

As a result, U1 received a score of 2, since both the spectrophotometric and HPLC methods can be used to assay the active ingredient. U4 received a score of 1, as only the HPLC method is suitable. U2 and U3 were assigned a score of 0, as none of the developed analytical methods were ideal for API quantification. Therefore, it is necessary to develop a new analytical method to enhance the assessment of API content. Additionally, alternative approaches, such as using an extraction method or selecting a different specific wavelength, may be considered, as the oils used to develop U2 and U3 interfered with the analyte.

#### 3.3.3. Mathematical Scoring Evaluation

**Figure 4:**
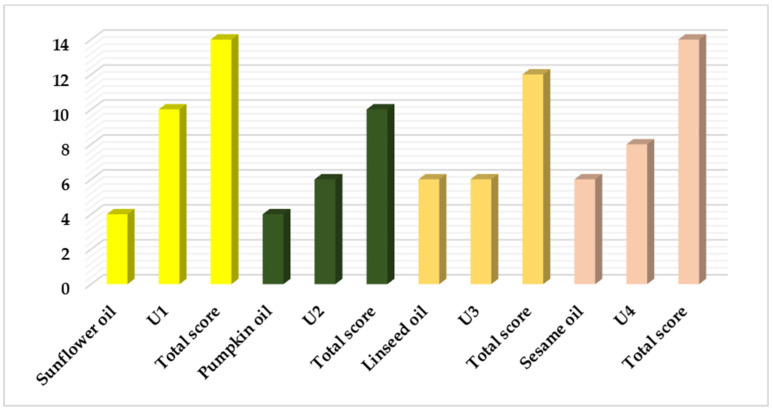
The scores obtained by the blank oils and the CBD oils, and the total score obtained by cumulating the unloaded oil score with the CBD-oil score.

As shown in Figure 4, the final scores indicate that sunflower oil and U1 had a total score equal to that of sesame oil and U4. This was then followed by linseed oil and pumpkin oil. To identify the most suitable CBD oil, the results for the different CBD oils were compared. In this scenario, U1 (with a score of 10—the perfect score) achieved better results than U4 (with a score of 8). Therefore, U1 was chosen as the lipophilic component for developing the CBD emulsions.

CBD content was further assessed using high-performance liquid chromatography (HPLC) at 208 nm. Over a three-month storage period, a gradual decline in CBD concentration was observed across all formulations. Sunflower oil exhibited the highest stability, with minimal loss. Sesame oil showed a 15% reduction, while linseed oil experienced a 22% decrease in CBD content. Notably, pumpkin seed oil showed a significant reduction, retaining only 22.8% of its initial CBD concentration after 3 months, indicating accelerated degradation or incompatibility with the cannabinoid. These findings highlight the importance of choosing oils characterised by low pro-oxidant components and advantageous fatty acid profiles, as these factors directly impact the stability of lipophilic compounds such as CBD.

### 3.4. Characterisation of Cannabidiol (CBD) in Oil-in-Water Emulsions

Since U1 demonstrated the highest mathematical score concerning both the blank oil and the matrix containing the active ingredient, it was subsequently selected for the development of CBD emulsions. These pharmaceutical formulations were evaluated based on their stability index, particle size and distribution, as well as active ingredient content and stability. The results will be detailed in the subsequent subsections. All the developed emulsions exhibited a white, milk-like colour and texture, with no signs of separation were observed after the preparation process was completed. To expand the applicability of CBD in oral formulations, heterogeneous systems (emulsions) were also developed using sunflower oil as the lipid phase.

#### 3.4.1. Emulsion Stability (Short-Term Study)

The stability index of the emulsions after 1, 2, and 24 h is documented in Table 8. The stability index was assessed at 1, 2, and 24 h after preparation. In the initial three cases, the automated method demonstrated superior stability at 1 h and 2 h post-preparation. A concentration exceeding 5% did not improve the stability of the emulsion; moreover, due to the low S% results observed with E_10%a_ after 1 h, the production of mortar emulsions containing 10% emulsifiers was discontinued. This decision was based on the previous three results, wherein the automated method yielded better stability indices. As observed, the stability index tended to decrease over time. Considering all three time points, E_4%a_ was identified as the most stable, particularly during the early phase of evaluation. Additionally, the stability of E_3%a_ and E_4%a_ was compared based on S% after 24 h. Because E_4%a_ showed higher stability, a 4% emulsifier concentration was further selected for inclusion of the active ingredient.

The most stable formulation was achieved using a 4% emulsifier mixture, which was then employed to prepare a 4.76% CBD-loaded oil-in-water (O/W) emulsion via magnetic stirring. To develop a more stable formulation with both microbiological and antioxidant properties, vitamin E (an antioxidant) and Cosgard (a preservative) were added to the most stable emulsion. Future studies will consider more extended time frames and cycling tests to strengthen the stability assessment of the chosen heterogeneous formulation, especially across different compositions that may improve the initial stability at the start of the evaluation.

#### 3.4.2. Emulsion Type Evaluation

Two complementary methods were used to establish the type of emulsion W/O or O/W.

**Figure 5:**
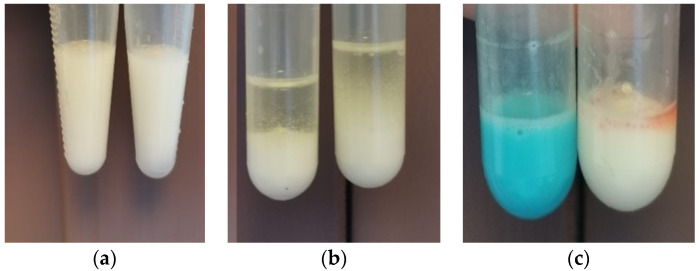
The emulsion type evaluation results through Method 1 (**a**,**b**) and Method 2 (**c**).

Although the emulsion was diluted with water, no changes were observed in its consistency; the formulation retained its original characteristics (Figure 5a). However, when oil was used as the diluent, a clear separation was observed (Figure 5b). The use of two colouring agents, methylene blue, a hydrophilic agent, and Sudan III, a lipophilic agent, showed that the hydrophilic agent dispersed readily within the emulsion. In contrast, Sudan III remained on the surface, forming particles that did not disperse in the emulsion (Figure 5c).

Both Method 1 and Method 2 produced consistent results, indicating that the resulting emulsion was of the oil-in-water (O/W) type. This provides a significant advantage from an administration perspective, as O/W emulsions are generally better tolerated by patients when taken orally.

#### 3.4.3. Particle Size and Distribution

**Figure 6:**
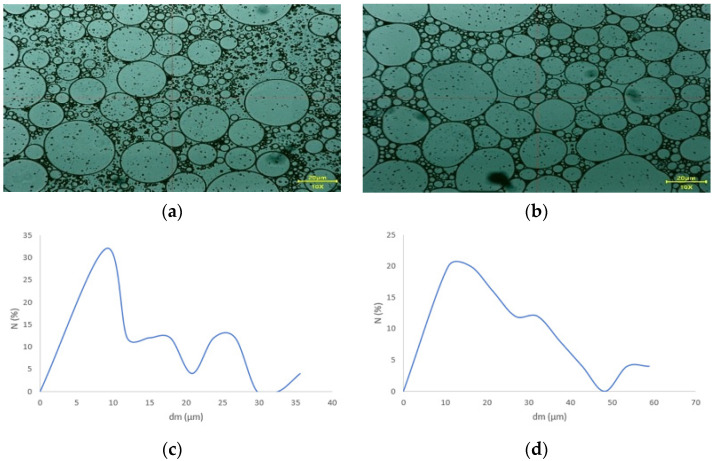
The microscopic evaluation of the oily droplets and the particle size distribution for the E_CBD_ (**a**,**c**) and E_blank_ (**b**,**d**).

The oily droplets from the proposed emulsions (E_CBD_ and E_blank_) are visible in Figure 6a,b. Since a heterogeneous pharmaceutical formulation had been developed, the particle size of the oil droplets can indicate the emulsion’s stability. For better comparison, an unloaded emulsion containing all ingredients except the active ingredient, and a loaded emulsion containing the active ingredient at a concentration of 4.76%, were prepared. Emulsions were obtained in both cases, as the emulsion without the active ingredient showed an average particle size of 10.26 µm (Figure 6c). In comparison, the CBD emulsion (E_CBD_) had a halved average particle size of 5.83 µm (Figure 6d). In practice, incorporating CBD into the heterogeneous matrix improved stability and reduced particle size.

### 3.5. API Content in the Emulsions

Since both the spectrophotometric and HPLC methods, as previously stated by Seccamani et al. and Patel et al. [31,32], were suitable for the U1, the CBD assay from the emulsion began with the spectrophotometric method. The results obtained through the spectrophotometric method are underscored in the following subsection.

#### 3.5.1. Spectrophotometric Determination of CBD from Emulsions

The average extracted CBD concentration was 14.18 µg/mL, compared to a theoretical value of 23.8 µg/mL, indicating approximately 59% recovery. The results emphasise the need for improved extraction techniques to address CBD retention in the lipophilic phase. The spectrophotometric method was suitable for quantifying active ingredients in the U1 formulation, which utilised sunflower oil due to its lower interference risk and simpler matrix. However, although U1 was incorporated into the heterogeneous formulation, the increased complexity of the matrix rendered the spectrophotometric assay unsuitable. The addition of emulsifiers Tween 80 and Span 80, vitamin E as an antioxidant, and Cosgard as a preservative heightened interference with the active ingredient assay. To address these limitations, the High-Performance Liquid Chromatography (HPLC) method was selected due to its superior selectivity and reliability.

#### 3.5.2. HPLC Quantification of CBD in Emulsions

Since the spectrophotometric methods did not closely match the theoretical values, the HPLC method was used. Because an emulsion is a more complex matrix than an oil-based formulation, different extraction techniques were employed. Two samples were tested and labelled P1 and P2. For the P1 sample, after collecting the emulsion, a 1:100 dilution in acetonitrile was prepared, followed by 60 min of magnetic stirring. The sample was diluted 1:20 and analysed by HPLC. For P2, the same dilutions were carried out, but no magnetic stirring was performed after the initial dilution. As shown in Table 9, the results were close to the theoretical concentration. Furthermore, the study showed that extra magnetic stirring during dilution before analysis did not significantly influence the extraction yield, whether for magnetically prepared or mortar-prepared emulsions. This suggests that the emulsified system enabled the sufficient release of CBD into the solvent phase, rendering these steps unnecessary for accurate analysis.

**Figure 7:**
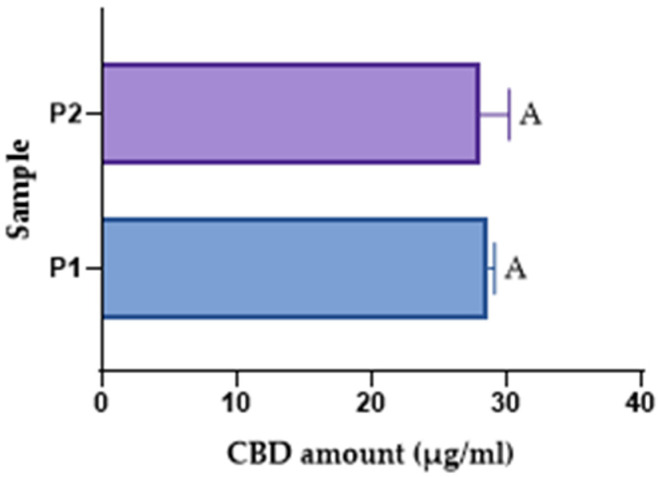
The *t*-test statistical evaluation of the extraction method.

To support the statement that the extraction method does not influence the amount of CBD assessed, a statistical test (*t*-test) was applied, revealing no statistically significant difference (fact expressed through the same letter-A) (Figure 7).

CBD quantification in the emulsion was performed using both UV spectrophotometry (at 210 nm) and HPLC (at 208 nm). The HPLC method showed greater precision and accuracy in determining the CBD content compared to UV analysis, which was more susceptible to matrix interference, particularly in the presence of surfactants and emulsified lipids. These findings are consistent with the literature, which recommends chromatographic methods over spectrophotometric methods for complex or turbid pharmaceutical systems [33,34].

When the CBD recovery was evaluated, it was determined that the extraction method was the issue; as a result, a switch from the spectrophotometric method to HPLC was necessary. Additionally, during emulsion evaluation, various methods and extractions were considered to determine whether the extraction process influenced the amount of API assayed. As previously mentioned, the extraction method did not significantly affect the amount of API extracted from the liquid matrix, although the results were higher than expected. This can be attributed to the emulsions, which are heterogenous formulations. Since the API is lipophilic, the amount of internal phase sampled may explain the observed increase in concentration. From previous experience with CBD formulations, when the matrix becomes more complex, the results from spectrophotometric assays tend to vary widely. Selectivity is very challenging to achieve with a spectrophotometric method for an API with a specific wavelength below 250 nm, as many excipients exhibit peaks in this range. The excipients in the formulation interfere with the active ingredient at the proposed wavelength; therefore, an HPLC method is necessary for a more accurate assay of the active ingredient.

The present study is constrained by the relatively brief stability monitoring duration of three months and the limited selection of lipid carriers, which comprised only commonly utilised edible plant-derived oils. Such limitations may not entirely capture the long-term physicochemical behaviour and oxidative stability of cannabidiol (CBD) formulations under diverse storage conditions. Future investigations will seek to extend stability assessments to 6–12 months, adhering to both long-term and accelerated ICH Q1A(R2) guidelines, and to broaden the spectrum of lipid excipients by integrating alternative natural oils with varied unsaturation and antioxidant characteristics, such as almond and jojoba oils, as well as fully or partially synthetic triglyceride-based carriers (medium-chain triglycerides). These strategies are anticipated to yield a greater understanding of the relationships among fatty acid composition, oxidative vulnerability, and CBD retention, thereby facilitating the development of robust, pharmaceutically suitable delivery systems.

### 3.6. Oral Versus Topical Application of the Developed Liquid Formulations

From a biopharmaceutical perspective, vehicle selection is critical because the oral bioavailability of CBD is intrinsically low, primarily due to limited aqueous solubility, intense first-pass metabolism, and high interindividual variability [35]. Oral absorption is relatively slow, with pharmacokinetic studies reporting a t_max_ of 1–6 h following ingestion [36]. In this context, identifying sunflower oil as a stable, analytically compatible vehicle is particularly relevant for ensuring dose uniformity and formulation robustness in oral products.

Topical administration, in contrast, is a well-established route in which CBD exhibits meaningful anti-inflammatory and analgesic effects [37]. Commercial formulations frequently employ ointment or cream bases, whose performance depends strongly on the delivery system. Emulsion-based systems can enhance cutaneous penetration through surfactants that reduce interfacial tension and increase skin permeability, thereby facilitating CBD release from the lipid matrix [38]. The O/W emulsion developed in this study, stabilised with an optimised Tween 80/Span 80 mixture, therefore provides a promising platform not only for oral applications but also for topical formulations requiring enhanced percutaneous transport.

### 3.7. Regulatory Considerations

From a regulatory standpoint, topical CBD products are governed by heterogeneous frameworks shaped by jurisdiction, product category, and plant-derived source of the ingredient. In the European Union, CBD may be incorporated into cosmetic products only when obtained from permitted plant parts and without therapeutic claims, as required by Regulation (EC) No. 1223/2009 [39]. Any reference to anti-inflammatory or analgesic effects reclassifies the product as a medicinal product, triggering compliance with Directive 2001/83/EC, GMP manufacturing, and clinical efficacy demonstration [40]. The Romanian market reflects these distinctions: many supplements or medical devices contain hemp seed or fibre extracts lacking pharmacological CBD concentrations, while certain topical or urological products may include low-level CBD provided that their primary mechanism of action is physical (e.g., barrier formation), in line with Regulation (EU) 2017/745 (MDR) [41].

In the United States, the FDA does not recognize CBD as an approved cosmetic or over-the-counter—OTC—active ingredient; therefore, any therapeutic claim places the product under the drug regulatory pathway (IND/NDA). Across major jurisdictions, manufacturers must ensure THC absence, excipient safety, and formulation stability according to the ICH Q1–Q6 quality guidelines [42]. The well-characterized emulsion systems proposed in this study align with these requirements and support the development of compliant, stable, and effective topical CBD formulations.

## 4. Conclusions

Several CBD oils (10% *w*/*w*) were formulated using various natural oils to assess stability indices over time, in accordance with pharmacopoeial standards. Two analytical methods were established to measure CBD in the proposed pharmaceutical matrices, each complying with ICH guidelines. When selectivity was not achieved with the spectrophotometric method, a previously validated HPLC method was verified and successfully employed. During stability testing, a decline in CBD content was observed, with sunflower oil demonstrating the best stability and pumpkin oil the lowest. After three months, fungal growth was found in the oils; therefore, the addition of a lipophilic preservative solution is recommended to improve their stability.

In addition, the lipid excipients selected in this study are Generally Recognised As Safe (GRAS) and compliant with Ph. Eur. standards, supporting their suitability for pharmaceutical and dermato-cosmetic use. Future work will extend stability testing to 6–12 months under ICH Q1A(R2) long-term and accelerated conditions to establish reliable shelf-life data, and will explore additional natural (e.g., almond, jojoba) and synthetic triglyceride-based vehicles. In most cases, decreasing particle size improves stability. Also, in the case of self-nanoemulsifying drug delivery systems SNEDDS stability is better, and this method is applied for substances that show a very low stability in liquid formulation and are prepared only when the patient is using the formulation. In conclusion, decreasing particle size or formulating SNEDDS increases stability. The results outlined in this study are preliminary and future advanced delivery strategies, such as nanoemulsions or (SNEDDS), will be investigated to enhance CBD protection, improve bioavailability and dermal penetration (if the external application is targeted), and ensure patient safety and regulatory compliance in the rapidly expanding CBD product market.

The selection of the oil matrix is of utmost importance in affecting both drug release and long-term stability. Sunflower oil has demonstrated optimal performance, balancing favourable physicochemical properties, high CBD release, and sustained stability over time. Emulsions offer an additional advantage for enhancing dispersion, ensuring dose uniformity, and improving patient compliance, particularly in dermato-cosmetic or oral applications.

Sunflower oil showed better stability mainly due to the balance between its fatty acid profile and natural antioxidant content, notably higher levels of tocopherols (primarily α-tocopherol). These act as natural antioxidants, reducing lipid oxidation and safeguarding sensitive compounds like CBD from oxidative degradation.

Sunflower oil contains a significant amount of oleic acid, a monounsaturated fatty acid that is more resistant to oxidation than polyunsaturated fatty acids, which means it forms fewer lipid peroxides during storage.

Chromatographic techniques, such as high-performance liquid chromatography (HPLC), remain the benchmark for quantifying cannabinoids within complex matrices. Simultaneously, UV spectrophotometry may be utilised in simpler systems where interference is minimal.

The effects of surfactant concentration and preparation methods on the properties of the CBD emulsion were studied. Since emulsions are heterogeneous mixtures, their stability is generally lower than that of oily solutions. The emulsion was of the O/W type with a small particle size, showing promising results that could be further improved to develop nanoemulsions with greater stability. Due to the complex composition of the emulsion, chromatographic methods were most suitable for measuring CBD. Minor differences were observed between the two preparation methods, indicating that emulsions can be produced by either method and yield similar particle sizes and stability.

In the future, additional ingredients such as antioxidants and various surfactants will be added to CBD oils and emulsions to enhance their stability.

## Figures and Tables

**Table 1:** The score repartition for the blank oils and CBD oils.

Parameters	Range 1	Score	Range 2	Score	Range 3	Score
Blank oils						
Density	±5%	2	±5.01–10%	1	>10%	0
Acid index	±10%	2	±10.01–15%	1	>15%	0
Saponification index	±10%	2	±10.01–15%	1	>15%	0
Peroxide index	±10%	2	±10.01–15%	1	>15%	0
Ester index	±10%	2	±10.01–15%	1	>15%	0
Maximum score		10				
CBD oils						
Assay method	Spectrophotometric	2	HPLC	1	None	0
Colour	Corresponds	2	Not corresponding	0		
Smell	Corresponds	2	Not corresponding	0		
Aspect	Corresponds	2	Not corresponding	0		
Stability study	3 months	2	2 months	1	1 month	0.5
Maximum score		10				

**Table 2:** The amount of ingredients used to develop the CBD emulsions.

Emulsion Codes/ Ingredients	CBD	Ultrapure Water	Sunflower Oil	Tween 80	Span 80	Vitamin E	Cosgard
	Mass (g)						
E_3%a_ E_3%m_	/	9.76	9.64	0.23	0.36		
E_4%a_ E_4%m_	/	9.69	9.52	0.31	0.48		
E_5%a_ E_5%m_	/	9.60	9.40	0.40	0.60		
E_10%a_ E_10%m_	/	9.20	9.20	0.80	1.20		
E_CBD_	4.76	9.58	9.41	0.31	0.48	0.02	0.2
E_blank_	/	11.96	11.79	0.31	0.48	0.02	0.2

**Table 3:** Preparation method and the amount of emulsifier used to obtain the heterogeneous formulation.

Code	Method Used	Emulsifier Concentration
E_3%m_	mortar trituration	3%
E_3%a_	magnetic stirring	3%
E_4%m_	mortar trituration	4%
E_4%a_	magnetic stirring	4%
E_5%m_	mortar trituration	5%
E_5%a_	magnetic stirring	5%
E_10%a_	magnetic stirring	10%

**Table 4:** Fatty oil density.

Oil Type	Density (g/mL)	Ph. Eur. 11 Stipulations Density (g/mL) [17]	% Deviation	Mathematical Scoring
Sunflower oil	0.891	0.920	2.94	2
Pumpkin oil	0.894	0.919	2.72	2
Linseed oil	0.889	0.928	3.99	2
Sesame oil	0.886	0.918	3.49	2

**Table 5:** The acid, saponification, ester, and peroxide values obtained for the selected oils and their mathematical scores.

Indexes	Sunflower Oil		Pumpkin Oil		Linseed Oil		Sesame Oil	
	Obtained	Ph. Eur. stip.	Obtained	Ph. Eur. stip.	Obtained	Ph. Eur. stip.	Obtained	Ph. Eur. stip.
Acid value	0.27 ± 0.02	≤0.6 mg KOH/g	4.22 ± 0.66	2–4 mg KOH/g	0.69 ± 0.14	≤4 mg KOH/g	0.83 ± 0.16	≤1.5 mg KOH/g
Score	2		2		2		2	
Saponification value	76.05 ± 10.73	188–194 mg KOH/g	98.39 ± 4.21	185–195 mg KOH/g	227.82 ± 1.18	189–197 mg KOH/g	87.21 ± 6.19	188–193 mg KOH/g
Score	0		0		0		0	
Ester value	75.78	187–193 mg KOH/g	94.17	181–191 mg KOH/g	227.13	185–193 mg KOH/g	86.38	187–192 mg KOH/g
Score	0		0		0		0	
Peroxide value	38.78 ± 0.84	≤10 meq O_2_/kg	22.06 ± 1.67	≤15 meq O_2_/kg	8.17 ± 0.82	≤15 meq O_2_/kg	3.77 ± 0.009	≤10 meq O_2_/kg
Score	0		0		2		2	
Total score	2		2		4		4	

**Table 6:** The spectrophotometric CBD assay and the % deviation from the theoretical concentration.

Oil Type	Determined Concentration (µg/mL)	Theoretical Concentration (µg/mL)	% Deviation
U1	86.47	89.10	−2.95
U2	123.94	88.92	+39.4
U3	70.51	89.38	−21.1
U4	53.44	88.56	−39.7

**Table 7:** The stability of the oils at 1 (T1), 2 (T2), and 3 (T3) months, and their method and stability score.

Oil Type	T1 (µg/mL)	T1 % Deviation	T2 (µg/mL)	T2 % Deviation	T3 (µg/mL)	T3 % Deviation	Theoretical (µg/mL)	Method Score	Stability Score
U1	48.44	+8.73%	47.30	+6.18%	45.48	+2.09%	44.55	2	2
U2	88.45	+99.0%	53.44	+20.2%	10.16	−77.1%	44.46	0	0
U3	66.39	+48.6%	60.94	+36.4%	34.97	−21.7%	44.69	0	0
U4	40.59	−8.3%	46.95	+6.0%	37.56	−15.2%	44.28	1	1

**Table 8:** The stability index for the selected formulations.

Emulsion Type	S% After 1 h	S% After 2 h	S% After 24 h
E_3%m_	79.50	34.43	15.98
E_3%a_	95.90	93.85	18.03
E_4%m_	71.07	42.14	17.35
E_4%a_	95.86	93.80	23.55
E_5%m_	62.5	52.08	41.66
E_5%a_	58.33	45.83	33.33
E_10%a_	60.86	47.82	19.56

**Table 9:** Concentration of CBD in emulsions determined by HPLC.

Sample	Mean Concentration (µg/mL)	Theoretical Concentration (µg/mL)
P1 (magnetic, shaken)	28.68 ± 1.72	23.8
P2 (magnetic, static)	28.00 ± 1.80	

## Data Availability

The original contributions presented in this study are included in the article/Supplementary Material. Further inquiries can be directed to the corresponding authors.

## Supplementary Materials

The following supporting information can be downloaded at: https://www.mdpi.com/article/10.3390/pharmaceutics17121533/s1, Figure S1: Linearity of the HPLC method used to assay the API from the CBD oils (U1-U4); Table S1: Main validation parameters of the HPLC method for cannabidiol determination; Figure S2: Selectivity of the HPLC method – comparison of blank oil (sesame oil) and CBD oil (U3).

## Abbreviations

The following abbreviations are used in this manuscript:

CBD	Cannabidiol
US	United States
HLB	Hydrophil–Lipophil Balance
O/W	Oil in water
O_2_	Oxygen
U/HPLC	Ultra/High Pressure Liquid Chromatography
W/W	Weight/weight
API	Active pharmaceutical ingredient
SD	Standard deviation
V/V	Volume/volume
Ph. Eur.	European Pharmacopoeia
