# Supplementary material for: Formulation and Analytical Evaluation of Liquid Cannabidiol Preparations: Comparative Study of Oil-Based Solutions and Emulsions
Source: Pharmaceutics. 2025 Nov 28;17(12):1533. doi: 10.3390/pharmaceutics17121533 (PMC12736438; doi:10.3390/pharmaceutics17121533)
Supplement: Supplementary file 1 [file pharmaceutics-17-01533-s001.zip › pharmaceutics-3998702-supplementary.pdf]

Supplementary material

Formulation and Analytical Evaluation of Liquid Cannabidiol Preparations: Comparative Study of Oil-Based Solutions and Emulsions

Robert-Alexandru Vlad [1], Lénárd Farczádi [2,*], Denisa Palișțan [1], Cezara Pintea [1,3], Paula Antonoaea [1], Emöke-Margit Rédai [1], Andrada Pintea [1,3], Cornelia-Titiana Cotoi [1], Adriana Ciurba [1], Magdalena Bîrsan [4,*] and Ruxandra-Emilia Ștefănescu [5]

1 Pharmaceutical Technology and Cosmetology Department, Faculty of Pharmacy, George Emil Palade University of Medicine, Pharmacy, Science and Technology of Targu Mures, 38th Gheorghe Marinescu Street, 540142 Targu Mures, Romania; robert.vlad@umfst.ro (R.-A.V.); denipalistan@gmail.com (D.P.); cezara.pintea@umfst.ro (C.P.); paula.antonoaea@umfst.ro (P.A.); emoke.redai@umfst.ro (E.-M.R.); andrada.pintea@umfst.ro (A.P.); titiana.cotoi@umfst.ro (C.-T.C.); adriana.ciurba@umfst.ro (A.C.)

2 Chromatography and Mass Spectrometry Laboratory, Centre for Advanced Medical and Pharmaceutical Research, George Emil Palade University of Medicine, Pharmacy, Science and Technology of Targu Mures, 38th Gheorghe Marinescu Street, 540142 Targu Mures, Romania

3 Medicine and Pharmacy Doctoral School, George Emil Palade University of Medicine, Pharmacy, Science, and Technology of Targu Mures, 38th Gheorghe Marinescu Street, 540142 Targu Mures, Romania

4 Department of Drug Industry and Pharmaceutical Biotechnology, Faculty of Pharmacy, "Grigore T. Popa" University of Medicine and Pharmacy from Iasi, 16 Universitatii Street, 700115 Iasi, Romania

5 Department of Pharmacognosy and Phytotherapy, Faculty of Pharmacy, George Emil Palade University of Medicine, Pharmacy, Science and Technology of Targu Mures, 38th Gheorghe Marinescu Street, 540142 Targu Mures, Romania; ruxandra.stefanescu@umfst.ro

* Correspondence: lenard.farczadi@umfst.ro (L.F.); magdalena.birsan@umfiasi.ro (M.B.)

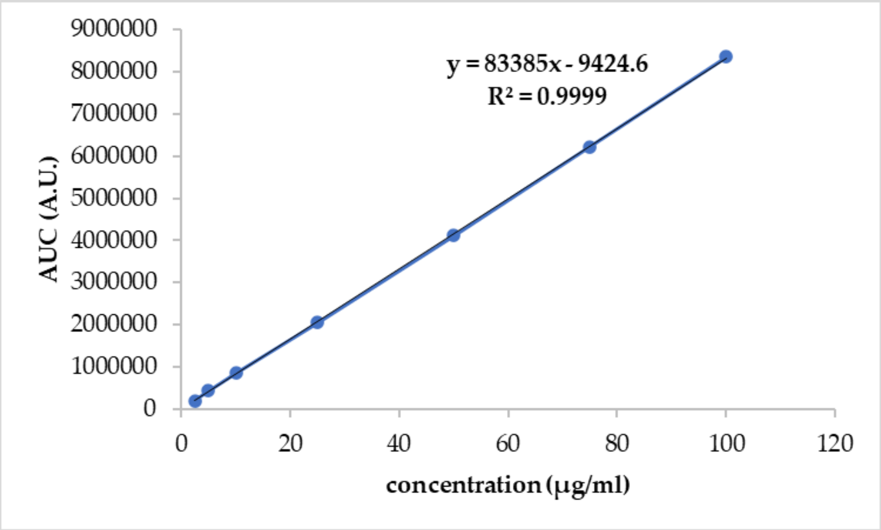

Figure S1. Linearity of the HPLC method used to assay the API from the CBD oils (U1-U4)

Table S1. Main validation parameters of the HPLC method for cannabidiol determination

Linearity	R = 0.9989-0.9999
Linearity range	1-100 µg/mL
Lower limit of quantification (LLOQ)	1 µg/mL, s/n=32
Carry-over	0.00%
Selectivity	99.80%
Precision (within-run)	0.98-1.51%
Accuracy (within-run)	95.58-101.42%

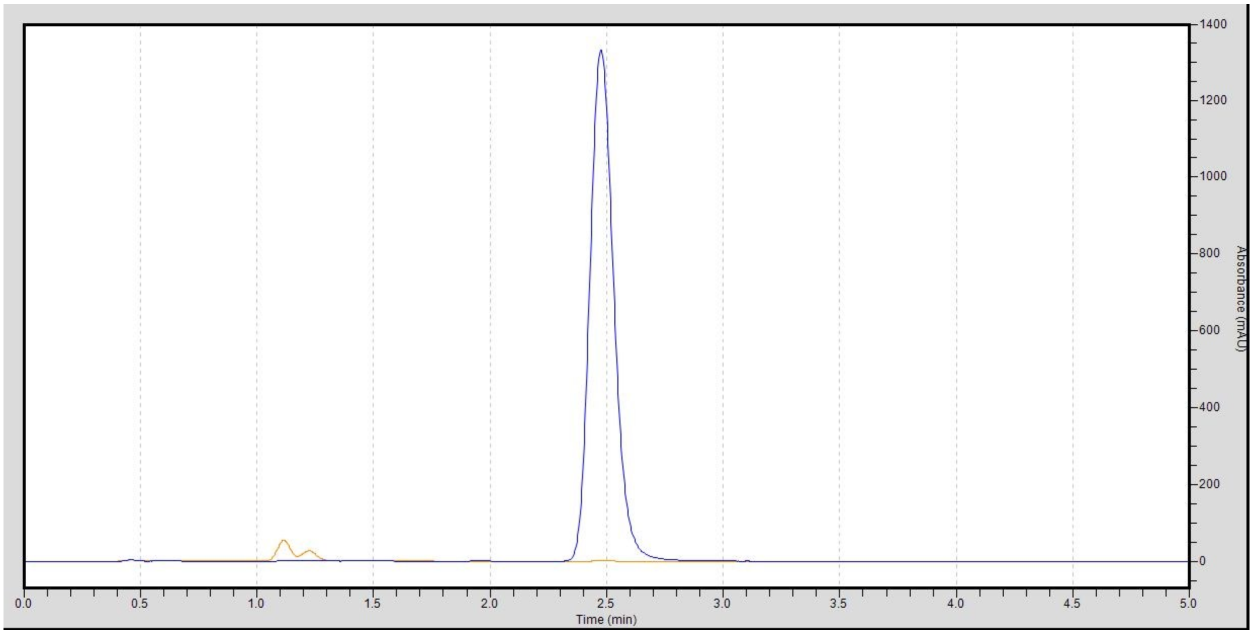

Figure S2. Selectivity of the HPLC method – comparison of blank oil (sesame oil) and CBD oil (U3)
